# Health Behaviors of Breast Cancer Survivors with Hypertension: A Propensity Analysis of KNHANES III-V (2005-2012)

**DOI:** 10.1371/journal.pone.0127346

**Published:** 2015-05-15

**Authors:** Ju-Ri Jeong, Sun Kim, So-Ra Jo, Ju-Youn Joh, Yeon-Pyo Kim

**Affiliations:** 1 Department of Family Medicine, Hwasun Chonnam National University Hospital, Hwasun-gun, Korea; 2 Clinic of Life After Cancer Treatment (LACT Clinic), Hwasun Chonnam National University Hospital, Hwasun-gun, Korea; University of Rochester, UNITED STATES

## Abstract

**Objective:**

This study examines health behaviors of breast cancer survivors with hypertension and compares them with those of non-cancer individuals with hypertension.

**Methods:**

In this cross-sectional study, a total of 10,996 hypertensive adults (≥ 19 years) who participated in the 2005-2012 Korean National Health and Nutrition Examination Survey (KNHANES) were considered. Data on alcohol consumption, smoking, physical activity, antihypertensive medication adherence, self-reported diet control, and sodium intake were collected through self-report questionnaire. A total of 64 breast cancer survivors with hypertension and 10,932 non-cancer participants with hypertension were identified. To better compare health behaviors of the two groups, 56 breast cancer survivors and 280 non-cancer participants were selected through the 1:5 nearest available matching based on estimated propensity scores. Multivariate analysis was conducted to determine any differences between the two groups.

**Results:**

According to multivariate analysis, breast cancer survivors with hypertension (n = 56) were significantly less likely to consume alcohol (odds ratio (OR): 3.75; 95% confidence interval (CI): 1.06-13.29) but significantly more likely to have sodium intake of more than 2400 mg (OR: 2.98; 95% CI: 1.27-6.97) than the propensity-matched control group (n = 280). There was no significant difference in other health behaviors between the two groups.

**Conclusions:**

Breast cancer survivors require active interventions for healthy behaviors related to the management of comorbid conditions such as hypertension to reduce the risk of cardiovascular disease and improve their overall survival rate.

## Introduction

Cancer, a leading cause of death in Korea [1], is a global medical issue. Advances in early-detection and treatment methods for cancer have led to a sharp increase in the number of people living with cancer [2]. Between 1999 and 2010, there were 960,654 cancer survivors in Korea. In 2014, nearly one million people are expected to be cancer survivors. According to previous research, five-year survival rates for thyroid cancer, breast cancer, colon cancer, and stomach cancer are 99.8%, 91.0%, 72.6%, and 67.0%, respectively [3]. Cancer survivors have been recognized as a growing group. Therefore, their health problems and quality of life have become a topic of considerable interest among physicians.

Although anticancer treatment and prevention methods for recurrence of primary cancer form the main focus of cancer survivor care, the most common cause of death among cancer survivors has ironically been cardiovascular disease (CVD), the same main cause as in the general population [4,5]. According to a retrospective cohort study using data from the 1992–2005 period in U.S., older women (≥ 66 years) diagnosed with breast cancer were almost equally likely to die from breast cancer as they were from CVD [5]. Hypertension, a well-known risk factor for CVD, is the most prevalent chronic condition among cancer survivors, regardless of the type of cancer. It can influence their prognosis and survival [6,7]. According to a nine-year historical cohort study in the U.S., hypertension has prognostic significance in the survival of breast cancer patients [8].

Because breast cancer incidence and mortality rates among women are closely related to their lifestyle [9], a number of studies have examined health behaviors of breast cancer survivors from a variety of data source. However, they produced mixed results [2,10–15]. To the best of our knowledge, no study has observed health behaviors of breast cancer survivors with a specific comorbidity compared to a proper control group in the literature. Therefore, the objective of this study was to compare health behaviors of breast cancer survivors with hypertension with those of non-cancer individuals with hypertension by using data from KNHANES (Korean National Health and Nutrition Examination Survey) 2005–2012. This will offer a starting point for research on health behaviors of breast cancer survivors with a specific comorbid condition.

## Materials and Methods

### Study population

Data were obtained from KNHANES III (2005), IV (2007–2009), and V (2010–12). KNHANES is a nationally representative cross-sectional survey conducted by the Korean Ministry of Health and Welfare and the Korea Centers for Disease Control and Prevention (KCDC). Individuals participating in KNHANES completed questionnaire consisting of a health interview survey, a health behavior survey, a nutrition survey, and a health examination survey. Further information can be found in “The 3^rd^~5^th^ (2005–2012) KNHANES Sample”, which are available on the KNHANES website ([16]; see S1 Dataset). The data from KNHANES is available on request by email if the applicant logs onto the “Korea National Health and Nutrition Examination Survey” website and specifies with annual reports he or she needs. Because the KNHANES data set was publicly available, the study protocol did not require any institutional review board approval. A total of 84,550 unweighted individuals participated in KNHANES III-V, including 34,145 in III (2005), 24,871 in IV (2007–2009), and 25,534 in V (2010–2012). In this study, data on 10,966 hypertensive adult (≥ 19 years old) participants were evaluated. Participants included 64 breast cancer survivors with hypertension and 10,932 non-cancer participants with hypertension. Before we estimated propensity score for matching, 8 breast cancer survivors with missing values of variables were excluded. The 1: 5 nearest available matching ratio based on estimated propensity scores between breast cancer survivors and non-cancer individuals was employed. Therefore, 56 breast cancer survivors with hypertension and 280 non-cancer controls with hypertension were obtained.

### Measures

#### Sociodemographic characteristics and chronic conditions

Sociodemographic characteristics and comorbid chronic conditions that may have significant effect on health behaviors were selected based on previous research [10–12,17]. Sociodemographic characteristics included age, gender, height, weight, waist, education (≤ elementary school, middle school, high school, and ≥ college), marital status (single, married, and widowed/divorced/separated), household income (low, middle-low, middle-high, and high), and private insurance. Comorbid chronic conditions used in this study included the following eight conditions related to seek health care services and contact to physicians: dyslipidemia, diabetes, ischemic heart disease, stroke, chronic respiratory disease (asthma and chronic obstructive lung disease), arthritis, thyroid disease, and depression. These conditions were considered present for those who had been diagnosed by a physician. Participants were classified as cancer survivors if they reported that they had been diagnosed by a physician with breast cancer. Their current cancer status could not be assessed because no data was collected in the KNHANES on cancer symptoms or cancer treatments.

#### Health behaviors

The following health behaviors were assessed: alcohol consumption (yes: at least once a month in the past year; or no: fewer than once a month in the past year), smoking (current smokers; or non-current smokers: those who never smoked and former smokers who quit smoking), self-reported diet control (yes: those who answered "yes" to a question "do you control your diet due to special reasons?"; or no), and physical activity (yes; or no). Physical activity was measured through the frequency (sessions per week) and duration (in minutes or hours) of each session. Cancer survivors were assigned to one of two physical activity groups according to the 2013 American Heart Association/the American College of Cardiology (AHA/ACC) guidelines [18]. Subjects were considered physically active if they participated in moderate-to-vigorous aerobic physical activity three to four sessions per week and the activity lasted an average of 40 minutes per session. Taking antihypertensive medication was measured by monthly frequency. A good antihypertensive medication adherence was defined as daily medication. Levels below this were regarded as not meeting sufficient medication adherence. Dietary sodium intake was obtained based on their 24-hour recall in the KNHANES nutrition survey. According to the 2013 AHA/ACC guidelines, hypertensive patients are recommended to reduce dietary sodium intake to no more than 2,400 mg (6 g sodium chloride) [18]. Sodium intakes below 2,400 mg were regarded as meeting the recommendation.

### Statistical analysis

All statistical analyses were conducted using SPSS complex sample procedure because KNHANES data set was selected through a representative, stratified, and clustered sampling method instead of a random sampling method. The KNHANES is providing the weight of individual participant of each survey, the stratification variables and the cluster variables. After creating the plan file by specifying these, all statistical analyses were conducted using SPSS complex sample procedure. Sociodemographic characteristics of participants were evaluated as un-weighted numbers and weighted percentages for categorical data. Means and standard error were used for continuous data. After propensity score matching, a logistic regression analysis was conducted to investigate any differences in health behaviors between breast cancer survivors and propensity-matched controls using two different approaches: unadjusted or multivariable-adjusted (entering age, height, weight, ischemic heart disease, arthritis, and significant covariate (*p* < 0.1) in univariate analysis). For a more accurate comparison, univariate analysis was conducted to select significant covariates. Only significant covariates (*p* < 0.1) were included in the adjustment. Because the KNHANES is a secondary data source, we provided the table including the variable name, the variable label, the original response scale, and how the response scale was recorded (see S1 Table). SPSS 22.0.0.1 (IBM Co, Armonk, NY, USA) was used in all statistical analyses. Statistical significance was considered when *p* value was less than 0.05. For propensity matching, we used propensity matching add-on for SPSS (Propensity score matching for SPSS, version 3.0.2).

### Assembly of study cohort: propensity score matching

Considering that the balance between two groups achieved by randomization may be lost, we used propensity score approach to assemble a cohort and control for any imbalance in confounding factors. The propensity score for an individual, defined as the conditional probability of being the case group given the individual’s covariates, can be used to balance covariates in the two groups to reduce this bias [19]. Propensity scores were calculated for each of the 56 cancer survivors and the 7173 non-cancer participants who were hypertensive adults without missing value of variables through multivariable logistic regression analysis based on baseline characteristics (Table 1) as covariates. Weights of individual participants that were provided by the Korean Ministry of Health and Welfare for the analysis of the KNHANES data were included in the covariates.

**Table 1:** Sociodemographic characteristics and comorbid conditions of study participants by their cancer history before and after 1: 5 propensity score matching (KNHANES III-V, 2005–2012).

Characteristic	Before matching		After matching	
	Cancer	Noncancer	Cancer	Noncancer
Study N	64	10932	56	280
Age (y)	64.2±1.4	61.5±1.4	64.5±1.4	65.7±1.7
Women	64(100)	6233(53.3)	56(100)	280(100)
Study weight	999.7±144.1	967.2±144.6	1027.8±146.8	891.5±156.3
Height (cm)	153.2±0.6	159.7±0.6	153.2±0.6	151.0±0.8
Weight (kg)	60.6±1.6	64.3±1.6	60.6±1.6	58.4±1.8
Waist (cm)	86.0±1.4	86.6±1.4	86.0±1.4	85.7±1.6
Education				
≤Elementary	38(64.4)	5564(48.3)	36(66.2)	193(66.8)
Middle school	8(12.3)	1563(15.1)	6(11.2)	35(12.2)
High school	13(17.1)	2266(23.6)	10(16.0)	44(18.1)
≥College	4(6.2)	1187(13.0)	4(6.5)	8(2.9)
Marital status				
Single	1(0.8)	186(2.7)	1(0.8)	4(1.4)
Married	37(59.7)	7736(71.9)	31(58.9)	154(55.3)
Widowed/divorced/separated	26(39.5)	2956(25.5)	24(40.3)	122(43.3)
Household income				
Low	25(34.5)	3906(32.4)	23(34.9)	119(40.4)
Middle-low	13(21.7)	2729(25.9)	10(20.7)	59(19.6)
Middle-high	12(17.3)	2121(20.9)	12(18.0)	57(21.8)
High	13(26.5)	1938(20.8)	11(26.5)	45(18.2)
Private insurance				
Yes	25(42.0)	4217(50.6)	33(58.0)	161(55.7)
No	33(58.0)	5057(49.4)	23(42.0)	119(44.3)
Comorbidity				
Dyslipidemia	19(31.8)	2032(21.6)	18(32.6)	80(29.8)
Diabetes	20(21.5)	2224(19.9)	15(19.9)	75(23.5)
Ischemic heart disease	5(9.3)	725(6.0)	5(9.8)	14(5.0)
Stroke	3(3.7)	763(6.1)	2(3.0)	11(3.4)
Chronic respiratory disease (Asthma,COPD*)	7(11.8)	643(5.2)	5(11.1)	22(6.5)
Arthritis	31(58.0)	3154(25.0)	28(58.5)	123(38.7)
Thyroid disease	6(7.0)	413(3.6)	5(7.1)	20(7.3)
Depression	9(16.4)	612(5.5)	9(16.4)	37(10.9)

Results were expressed as un-weighted numbers (weighted %) or mean ± SE (standard error).

*COPD: Chronic obstructive pulmonary disease

**Fig 1:**
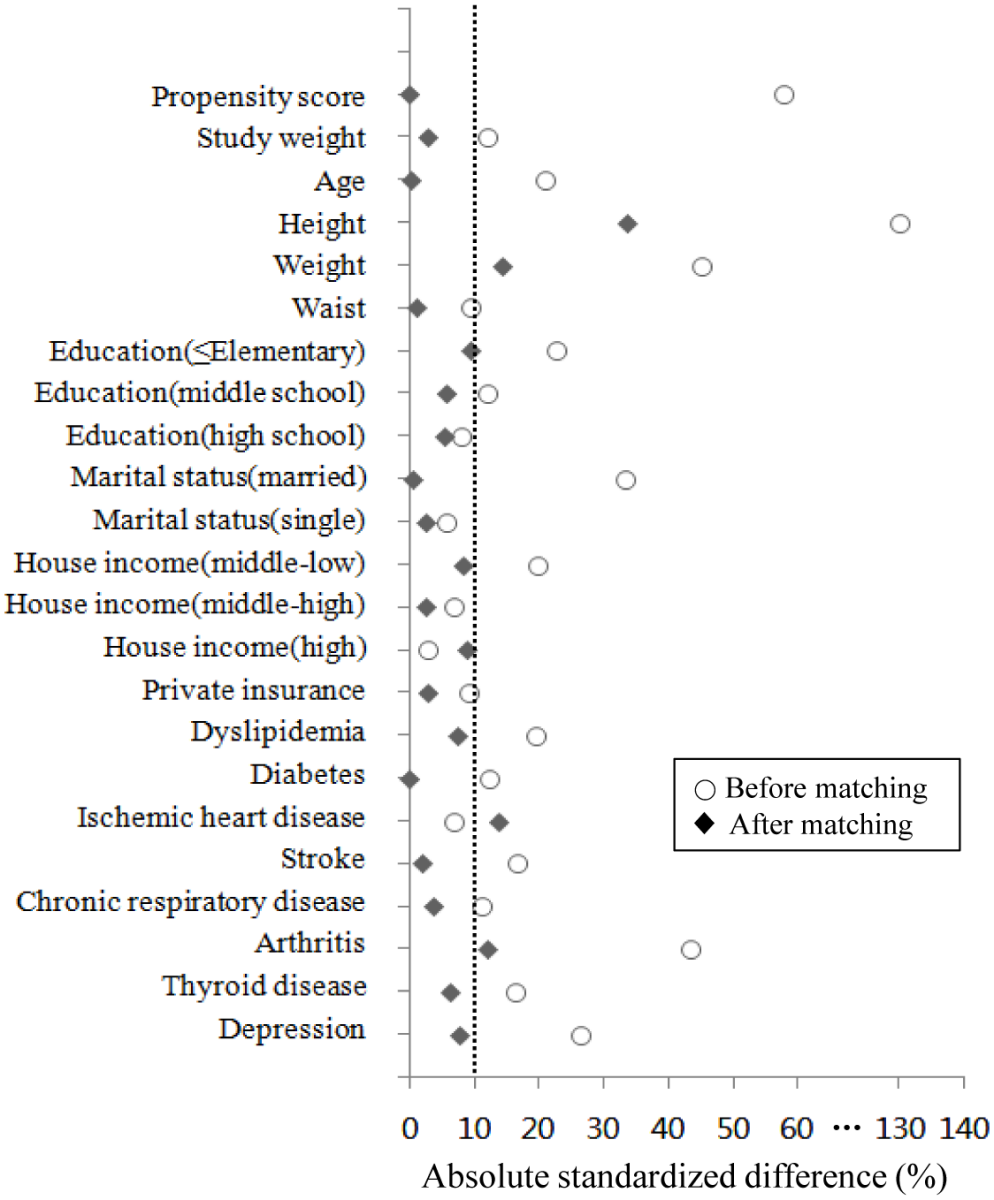
Absolute standardized differences. Absolute standardized differences in baseline characteristics of breast cancer survivors with hypertension compared to non-cancer participants with hypertension before and after 1:5 propensity score matching. Y axis was baseline characteristics. X axis was the percentage of absoluate difference “before” or “after” matching.

After estimating the propensity score, our sample sizes of the cancer and noncancer participants varies greatly. Therefore, one to five matching ratio was used [20]. The nearest available matching based on estimated propensity scores was performed with an application program in SPSS to select the most similar propensity score across the groups in a 1:5 ratio [19–21]. Exact matching on gender was used. Absolute standardized differences were estimated to evaluate pre-match imbalance and post-match balance. Results were presented as a Love plot (Fig 1) [22]. An absolute standardized difference of 0% indicated no residual bias. Differences less than 10% were considered inconsequential [23]. Because substantial differences in covariates (height, weight, ischemic heart disease, and arthritis) between matched participants remained after matching, we conducted additional regression adjustments to reduce such differences [24].

## Results

### Characteristics of cancer survivors

The sociodemographic characteristics and comorbid conditions of cancer survivors and non-cancer controls before and after 1:5 propensity score matching are summarized in Table 1. Before matching, 8 breast cancer survivors with missing values of variables among the 64 breast cancer survivors were excluded. The 56 breast cancer survivors were all female with mean (±SE) age of 64.5 ± 1.4 years. Their mean waist was 86.0 ± 1.4 cm. CVD risk factors included dyslipidemia (32.6%) and diabetes (19.9%). A history of ischemic heart disease and stroke was reported by 9.8% and 3.0% of all cancer survivors, respectively. After the matching procedure, breast cancer survivors with hypertension and non-cancer participants with hypertension were more similar to each other with regards to all 18 baseline covariates compared to pre-matching (Table 1 and Fig 1). Our propensity score matching reduced standardized differences for most observed covariates below 10% in absolute values, demonstrating substantial improvement in covariate balance across the groups, except height, weight, ischemic heart disease, and arthritis (Fig 1).

### Health behaviors of cancer survivors and non-cancer controls

**Fig 2:**
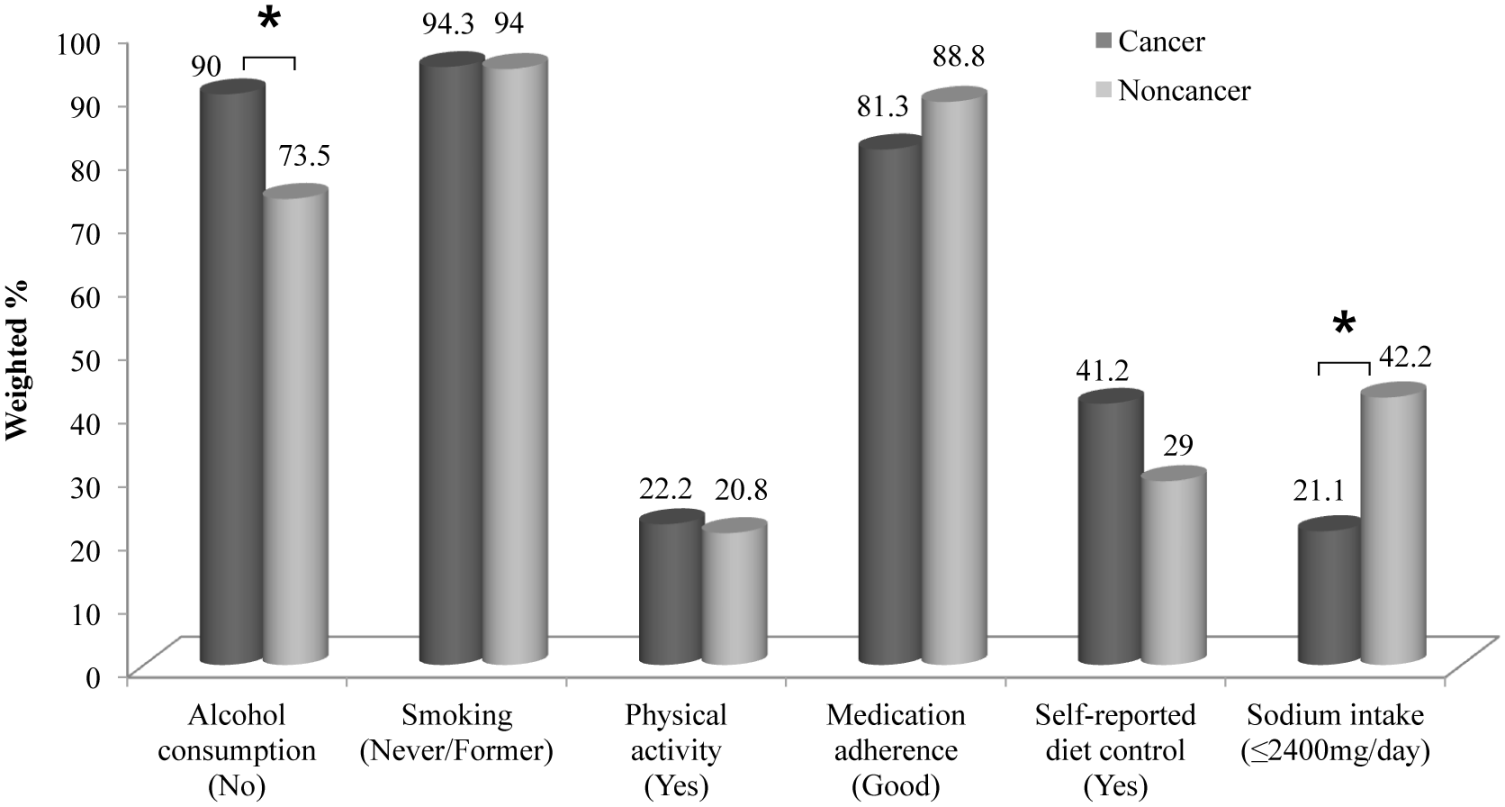
A comparison of health behaviors between breast cancer survivors and propensity-matched participants. The group of breast cancer survivors with hypertension was significantly less likely to consume alcohol but more likely to have sodium intake of more than 2,400 mg than the propensity-matched control group. *: *p* < 0.05.

**Fig 3:**
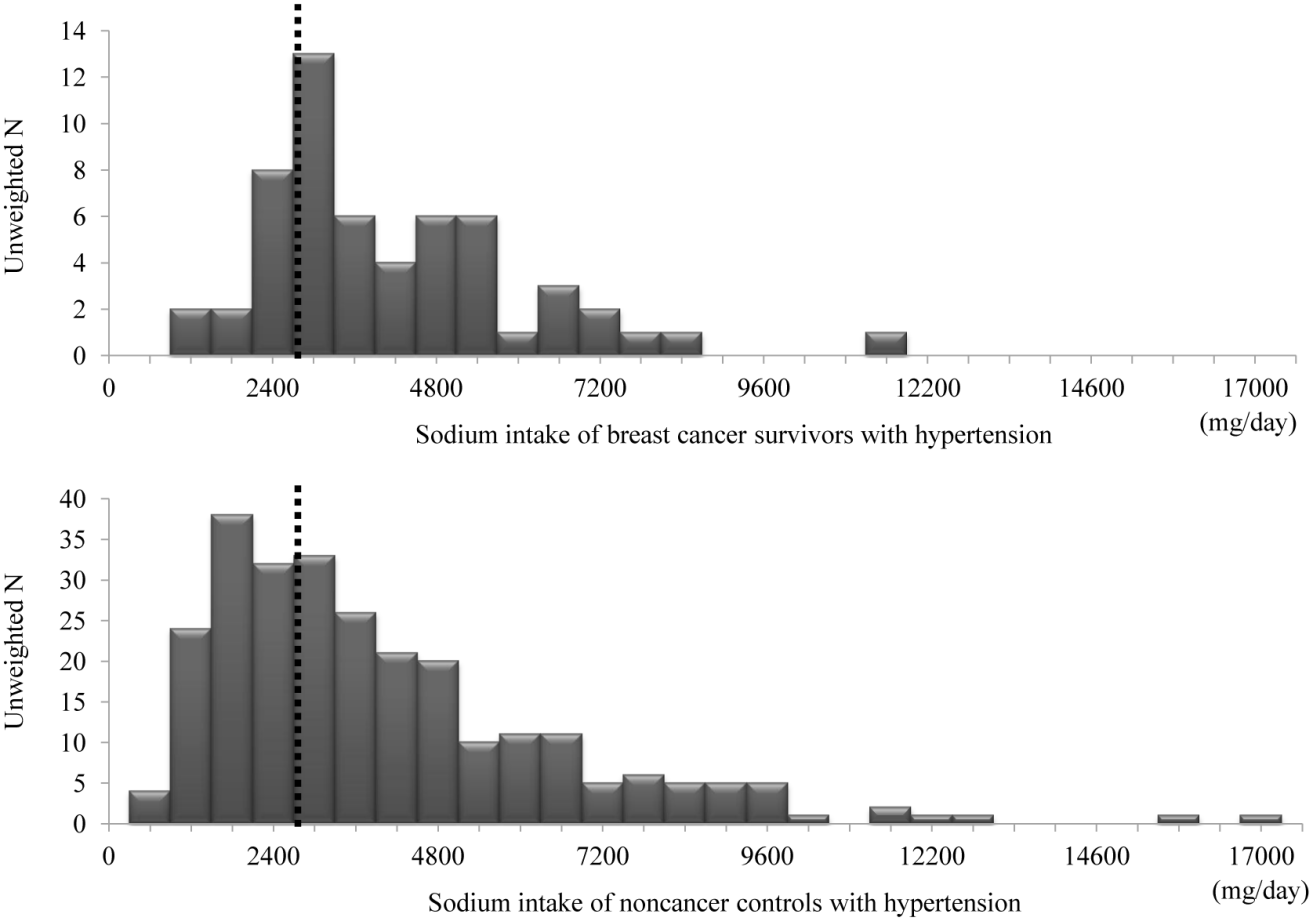
Sodium intake distribution of breast cancer survivors and propensity-matched participants. In the group of breast cancer survivors with hypertension, only 12 (21.1%) reported an intake of less than 2,400 mg sodium per day. A total of 44 (78.9%) reported a sodium intake of more than 2,400 mg per day. The breast cancer survivor group was significantly more likely to have sodium intake of more than 2,400 mg than the propensity-matched control group.

Multivariate logistic regression analysis revealed significant differences in alcohol consumption and sodium intake between the group of breast cancer survivors with hypertension and the control group. Breast cancer survivors with hypertension (n = 56) were significantly less likely to consume alcohol (OR: 3.75; 95% CI: 1.06–13.29) but significantly more likely to have sodium intake of more than 2,400 mg (OR: 2.98; 95% CI: 1.27–6.97) than the propensity-matched control group (n = 280). There was no significant differences in terms of smoking, physical activity, antihypertensive medication adherence, or self-reported diet control (Table 2 and Fig 2). Sodium intake distribution of propensity matched participants was shown in Fig 3.

**Table 2:** A comparison of health behaviors between breast cancer survivors and propensity-matched participants (KNHANES III-V, 2005–2012).

Variables	Cancer	Noncancer	Unadjusted OR^1^ (95% CI)	P-Value	Adjusted OR^2^ (95% CI)	P-Value
	N (%)	N (%)				
Study N	56(100)	280(100)				
Alcohol consumption						
No	52(90.0)	214(73.5)	3.25(0.91–11.69)	0.071	3.75(1.06–13.29)	0.040
Yes	4(10.0)	64(26.5)	Reference		Reference	
Smoking status						
Never/Former	52(94.3)	264(94.0)	1.07(0.30–3.80)	0.920	1.29(0.34–4.95)	0.711
Current	4(5.7)	15(6.0)	Reference		Reference	
Physical activity						
Yes	9(22.2)	56(20.8)	1.09(0.41–2.87)	0.867	0.90(0.33–2.44)	0.830
No	47(77.8)	222(79.2)	Reference		Reference	
Medication adherence						
Good	51(81.3)	247(88.8)	0.55(0.17–1.76)	0.310	0.54(0.19–1.54)	0.251
Poor	5(18.7)	33(11.2)	Reference		Reference	
Self- reported diet control						
Yes	23(41.2)	80(29.0)	1.72(0.80–3.70)	0.168	1.78(0.81–3.92)	0.150
No	33(58.8)	182(71.0)	Reference		Reference	
Sodium intake						
≤2400mg/day	12(21.1)	98(42.2)	Reference		Reference	
>2400mg/day	44(78.9)	165(57.8)	2.73(1.19–6.27)	0.018	2.98(1.27–6.97)	0.012

Results were expressed as un-weighted numbers (weighted %)

Logistic regression models incorporated sampling weights.

^1^ Unadjusted;

^2^ Adjusted for age, height, weight, ischemic heart disease, arthritis and significant covariates (*p* ≤ 0.1) from the univariate analysis.

OR: Odds ratio; 95% CI: 95% confidence interval

## Discussion

This is the first comparison between health behaviors of breast cancer survivors with hypertension and non-cancer controls with hypertension. Our results revealed that breast cancer survivors with hypertension were significantly less likely to consume alcohol but more likely to have sodium intake of more than 2400 mg. No significant difference in other health behaviors was found between the two groups. Contrary to our expectations, a substantial portion of breast cancer survivors with hypertension did not practice healthy behaviors.

The cancer survivor group was significantly less likely to consume alcohol than the control group (10.0% vs. 26.5%). Although any direct comparison of studies on alcohol consumption of breast cancer survivors should be made with caution because definitions of study participants' alcohol consumption and cancer status tend to vary across studies, the rate of alcohol consumption found in the present study is lower than that generally indicated in previous studies [10,11]. One study found that 51% of early-stage breast cancer survivors were current drinkers (0.5 g/day of alcohol) [11]. Another study reported that 14% of African-American breast cancer survivors were regular drinkers [10]. However, there is controversy over alcohol consumption among breast cancer survivors. Heavy alcohol consumption is generally associated with increased risk of breast cancer recurrence. On the other hand, limited alcohol drinking is associated with a possible reduction of mortality not related to breast cancer due to possible cardio-protective effects of alcohol [11,25]. These results suggest that physicians should advise breast cancer survivors with hypertension to practice moderate alcohol consumption. Cancer type and comorbid conditions should be included in making recommendations to patients on alcohol consumption [26].

The proportion of breast cancer survivors with hypertension who reported to be current smokers at the time of participation was 5.7%. The proportion of current smokers in participants who had no history of cancer was 6.0%. Our analyses showed that there was no significant difference with respect to current smoking status between the two groups. The ratio of current smokers among breast cancer survivors in the present study was lower than that in previous studies [2,11,14]. Therefore, the smoking rate in our study might be underestimated. A study in Korea reported that many cancer patients continue to smoke after their cancer diagnosis. A majority of them experience feelings of guilt and censure that can lead to their concealment of their smoking status from family members or healthcare professionals [27]. There is strong evidence that smoking is associated with a poor response to cancer treatment and increased risk of cancer recurrence, secondary cancer, and mortality from cardiovascular disease [14,28]. This suggests the importance of accurate smoking status in breast cancer survivors with hypertension. Because an individual’s cancer diagnosis alone may not be enough to cause a sustained improvements in his or her lifestyle including smoking cessation [29], physicians should implement effective intervention methods to help current smokers quit.

Only 22.2% of breast cancer survivors with hypertension met the recommended level of physical activity, which is inconsistent with findings of previous studies. This might be due to differences in standards for recommended levels of physical activity across studies. One study reported that 69% of breast cancer survivors practiced 30 minutes brisk walking more than once a week [13]. Another study found that 47% of breast cancer survivors followed the physical activity recommendation of women’s health initiative in the U.S. [10]. The low level of physical activity in the present study may be explained by the use of stricter standards based on the updated 2013 AHA/ACC guidelines [18]. All current antihypertensive treatment guidelines emphasize the role of non-pharmacologic intervention methods, including physical activity [30]. Physical activity is closely associated with improvements in the quality of life. It can reduce the risk of death from breast cancer [12,13]. Although cancer survivors may face various obstacles to physical activity such as cancer pain, treatment complications, and motor disability, physicians should encourage patients to avoid inactivity and recommend personalized physical activity programs for each cancer survivor.

An overwhelming majority of both cancer survivors (81.3%) and non-cancer controls (88.8%) reported taking daily antihypertensive medication, which is consistent with findings of a previous study [31]. Significant variations across different groups of cancer survivors according to the type of cancer, age, income level, residential area, and duration of antihypertensive medication were observed [31]. In consistent with our results, they also found that breast cancer survivors' medication adherence was similar to that of the general population [31]. Because adequate pharmacologic treatment can manage hypertension effectively [32], physicians should assess and improve medication adherence.

Although breast cancer survivors reported to have more self-control of their diet than the controls (41.2% vs 29.0%), 78.9% of breast cancer survivors had sodium intake of more than 2400 mg in their 24-hour recall in the KNHANES nutrition survey (Table 2 and Fig 3), which was in consistent with the findings of previous studies [15,33] (Fig 2). Although there are many research and controversy about the association between higher sodium intake and stomach cancer [34,35], the effect of sodium on breast cancer has not been established. Few previous studies suggested that higher sodium intake might be associated with higher risk of breast cancer [36,37]. One study showed that an ‘unhealthy’ food consumption pattern including salty foods was associated with a significantly increased risk of breast cancer [36]. However, another study found that a history of breast cancer in women was associated with increased sodium excretion [37]. Based on these studies and our result, a diet pattern including higher sodium intake before breast cancer diagnosis might be continued after breast cancer diagnosis. The relatively high overall level of salt contributes to cancer risk indirectly by promoting metabolic syndrome and reducing the potassium/sodium ratio in the diet, both of which are thought to increase the risks of various cancers including breast cancer [38,39]. Characterizing the sodium intake before and after diagnosis may be more useful in determining the course of change for breast cancer survivors with hypertension. Because sodium is known as an important nutrient in blood pressure control, life modifications including lower sodium intake (< 2,400 mg per day) could reduce BP, enhance antihypertensive drug efficacy, and decrease risk of stroke and cardiovascular risk [18,40]. High intakes of salt-preserved foods and of salt probably have been shown to increase the risk of gastric cancer [34]. Therefore, there is a clear need for intervention methods to focus on healthy sodium intake for hypertensive breast cancer survivors to reduce cardiovascular risk and secondary cancer.

This study contributes to the literature in several ways. First, unlike previous studies including a large number of cancer sites, this study investigated health behaviors of survivors of a specific type of cancer, namely breast cancer, with a specific comorbid condition, namely hypertension. Previous studies have not reflected differences in health behaviors of cancer survivors according to the type of cancer. This study is part of our ongoing research program. Our results are inconsistent with the findings of a study focusing on gastric cancer survivors under the same program. Second, the present study is not hospital-based but community based study using KNHANES. Therefore, participants recruited may be considered as representatives of the Korea population to reflect real-world practices with no selection bias. Third, propensity score matching, an efficient and useful way to conduct a matched case control study based on a large cohort, was employed to increase the robustness of observed outcomes [41]. This study's quasi-randomized experiment allowed unbiased estimates based on the comparison of health behaviors between the two groups [19].

This study has some limitations derived from the use of KNHANES data to examine health behaviors. First, the analysis was based on self-report questionnaire except for several measurements. Self-reported data have some disadvantages in terms of potential labeling errors, recall bias, and a lack of precision in answers. In this regard, the results may underestimate smoking and alcohol consumption but overestimate other variables as a result of social desirability [42]. Second, the cross-sectional nature of data prevented the identification of health behaviors before the diagnosis of cancer. Therefore, the effects of this diagnosis on health behaviors of breast cancer survivors could not be examined. Third, the KNHANES did not include information on cancer care status. Thus, we were unable to determine the proportion of cancer survivors who were actively dealing with treatment or recurrent/advanced disease versus those living with disease and/or were symptom free. This point also raises the possibility that the physical activity findings were skewed toward inactivity if cancer survivors were actively dealing with their disease. Fourth, the results are based only on a single ethnic group (Koreans). Therefore, their generalization to other groups is limited. Fifth, the analysis employed a small sample (56 breast cancer survivors). In this regard, future research should employ a larger sample for more robust findings on health behaviors of breast cancer survivors with hypertension. Sixth, currently there is no propensity score analysis program that can be used to directly support analysis of complex samples. Although we conducted the propensity matching using propensity matching add-on for SPSS, the cases were matched without addressing the complex sampling design.

Although many breast cancer survivors are expected and reported to practice healthy behaviors because of their closer attention to enhancing their health and visiting physicians more frequently [43], a substantial proportion of breast cancer survivors with hypertension in the present study did not engage in healthy behaviors. The management of patients after their cancer diagnosis generally focuses on the cancer itself [43]. Therefore, any patient care programs for cancer survivors should use an integrated management strategy for cancer and comorbid conditions.

In conclusion, breast cancer survivors require active interventions for healthy behaviors related to the management of comorbid conditions such as hypertension to reduce the risk of cardiovascular disease and improve their overall survival rate.

## Supporting Information

S1 Dataset Detailed information about the variables used. Because the KNHANES is a secondary data source, we provided the table including the variable name (as specified in the KNHANES), variable label, original response scale, and how the response scale was recorded.
(ZIP)

S1 Table The Korea National Health and Nutrition Examination Survey (KNHANES) 2005–2012 provided as SPSS Data Format. (DOCX)
