# Supplementary material for: Health Behaviors of Breast Cancer Survivors with Hypertension: A Propensity Analysis of KNHANES III-V (2005-2012)
Source: PLoS One. 2015 May 15;10(5):e0127346. doi: 10.1371/journal.pone.0127346 (PMC4433251; doi:10.1371/journal.pone.0127346)
Supplement: S1 Table — (DOCX) [file pone.0127346.s002.docx]

**S1 Table**. Detailed information about the variables used

| **Variable label** | **Variable name**  **(as specified in the KNHANES)** | **Original response scale** | **How the response scale was recoded** |
| --- | --- | --- | --- |
| Hypertension | HE_HPdg(4^th^,5^th^)  DI1_dg(3^rd^) | Whether hypertension was diagnosed by a physicians (4^th^,5^th^)  0.No 1.Yes  Whether hypertension was diagnosed by a physicians (3^rd^)  1.Yes 0.No 8.Non-applicable 9.Don’t know | 1.Yes: A participants answered, “yes”  0.No: Remainder. |
| Breast cancer | DC4_dg(4^th^,5^th^)  DC4_lt(3^rd^) | Whether a breast cancer was diagnosed by a physicians (4^th^,5^th^)  1.Yes 0.No  Whether a breast cancer was diagnosed by a physicians (3^rd^)  1.Yes 0.No 8.Non-applicable(men) 9.Don’t know | 1.Yes: A participants answered, “yes”  0.No: Remainder. |
| Age | Age | Age | Use as is. |
| Gender | Sex | Gender  1.Men 2.Women | Use as is. |
| Height | HE_ht (4^th^,5^th^)  HE_HT(3^rd^) | Height(cm) | Use as is. |
| Weight | HE_wt(4^th^,5^th^)  HE_WT(3^rd^) | Weight(kg) | Use as is. |
| Waist | HE_wc(4^th^,5^th^)  HE_WC(3^rd^) | Waist(cm) | Use as is. |
| Education | Educ | Education(4^th^,5^th^)  1.Village school 2.Iliteracy 3.Elementary school 4.Middle school 5.High school 6.2/3year college 7.4yrear college 8.Graduate school 88. Non-applicable 99. Don’t know  Education(3^rd^)  0.Preschool 1.Iliteracy 2. Elementary school 3. Middle school 4. High school 5.College 6. Graduate school 9. Don’t know | 1. ≤Elementary: A participants answered, “Xitang, Iliteracy, Preschool, or Elementary school”  2. Middle school: A participants answered, “middle school”  3. High school: A participants answered, “high school”  4. ≥College: A participants answered, “2/3year college, 4yrear college, or Graduate school” |
| Marital status | Marri_2(4^th^,5^th^)  Marri(3^rd^) | Marital history(4^th^,5^th^)  1.Married 2.Single 3.Don’t know  Marital status(4^th^,5^th^)  1.Sponse,live together 2.Sponse,seperated 3.Widowed  4.Divorced 8.Response by deny  9.Don’t know 88.Non-applicable 99.No response  Marital status (3^rd^)  1.Single 2.Sponse 3.Widowed 4.Divorced 5.Seperated 8.Non-applicable 9. Don’t know | 1.Single: A participants answered, “single”  2.Married: A participants answered, “Sponse, live together(4^th^,5^th^)or Sponse(3^rd^)”  3.Widowed/divorced/separated: A participants answered, “Sponse,seperated(4^th^,5^th^), Separated,Widowed, or Divorced” |
| Household income | Ho_incm | House income (4^th^,5^th^)  1.low 2.Middle-low 3.Middle-high 4.High  Income quartile (house) (3^rd^)  1.1^st^ quartile 2.2^nd^ quartile 3.3^rd^ quartile 4.4^th^ quartile | 1.low or 1^st^ quartile  2.Middle-low or 2^nd^ quartile  3.Middle-high or 3^rd^ quartile  4.High or 4^th^ quartile |
| Private insurance | Npins(4^th^,5^th^)  HO_pins(3^rd^) | Private health insurance membership (4^th^,5^th^)  1.Yes 2.No 9.Don’t know 99.No response  Private health insurance membership (3^rd^)  1.Yes 0.No 9.Don’t know | 1.Yes: A participants answered, “yes”  0.No: A participants answered, “no or don’t know” |
| Dyslipidemia | DI2_dg | Whether a dyslipidemia was diagnosed by a physicians (4^th^,5^th^)  1.Yes 0.No 8.Non-applicable  Whether a dyslipidemia was diagnosed by a physicians (3^rd^)  1.Yes 0.No 8.Non-applicable 9.Don’t know | 1.Yes: A participants answered, “yes”  0.No: Remainder. |
| Diabetes | DE1_dg(4^th^,5^th^)  DE1_1(3^rd^) | Whether a diabetes was diagnosed by a physicians (3^rd^,4^th^,5^th^)  1.Yes 0.No 8.Non-applicable 9.Don’t know | 1.Yes: A participants answered, “yes”  0.No: Remainder. |
| Ischemic heart disease | DI4_dg | Whether a myocardial infarction or angina was diagnosed by a physicians (4^th^,5^th^)  1.Yes 0.No 8.Non-applicable  Whether a myocardial infarction or angina was diagnosed by a physicians (3^rd^)  1.Yes 0.No 8.Non-applicable 9.Don’t know | 1.Yes: A participants answered, “yes”  0.No: Remainder. |
| Stroke | DI3_dg | Whether a stroke was diagnosed by a physicians (3^rd^)  1.Yes 0.No 8.Non-applicable 9.Don’t know  Whether a stroke was diagnosed by a physicians (4^th^,5^th^)  1.Yes 0.No 8.Non-applicable | 1.Yes: A participants answered, “yes”  0.No: Remainder. |
| Chronic respiratory disease |  |  |  |
| COPD | HE_PFTdr(4^th^,5^th^)  DJ5_dg(3^rd^) | Whether a chronic obstructive pulmonary disease was diagnosed by a physicians (3^rd^,4^th^,5^th^)  0.No 1.Yes 8.Non-applicable 9.Don’t know | 1.Yes: A participants answered, “yes”  0.No: Remainder. |
| Asthma | DJ4_dg | Whether a asthma was diagnosed by a physicians (3^rd^,4^th^,5^th^)  0.No 1.Yes 8.Non-applicable 9.Don’t know | 1.Yes: A participants answered, “yes”  0.No: Remainder. |
| Arthritis | DM1_dg | Whether a osteoarthritis or rheumatoid arthritis was diagnosed by a physicians (4^th^,5^th^)  0.No 1.Yes 8.Non-applicable  Whether a osteoarthritis or rheumatoid arthritis was diagnosed by a physicians (3^rd^)  0.No 1.Yes 8.Non-applicable 9.Don’t know | 1.Yes: A participants answered, “yes”  0.No: Remainder. |
| Thyroid disease | DE2_dg | Whether a thyroid disease was diagnosed by a physicians (4^th^,5^th^)  0.No 1.Yes 8.Non-applicable  Whether a thyroid disease was diagnosed by a physicians (3^rd^)  0.No 1.Yes 8.Non-applicable 9.Don’t know | 1.Yes: A participants answered, “yes”  0.No: Remainder. |
| Depression | DF2_dg(4^th^,5^th^)  BP5(3^rd^) | Whether a depression was diagnosed by a physicians (3^rd^,4^th^,5^th^)  1.Yes 0.No 9.Don’t know | 1.Yes: A participants answered, “yes”  0.No: Remainder. |
| Alcohol consumption | Dr_month(4^th^,5^th^)  BD1_11(3^rd^) | Adults usually drunk (3^rd^)  1.fewer than once a month  2.at least once a month 3. Non-applicable  Monthly drinking(4^th^,5^th^)  0.fewer than once a month in the past year 1.at least once a month in the past year | 1.Yes: fewer than once a month(3^rd^)or fewer than once a month in the past (4^th^,5^th^)  0.No: at least once a month(3^rd^)or at least once a month in the past year(4^th^,5^th^) |
| Smoking status | BS3_1 | Adults current smoking(3^rd^,4^th^,5^th^)  1.current daily smoking 2.sometimes smoking 3.former smoker who quit smoking 8.Non-applicable 9.Don’t know | 1.Current: current daily smoking or sometimes smoking  0.Never/Former: former smoker who quit smoking, Non-applicable or Don’t know |
| Physical activity | BE3_21*BE3_22  BE3_11*BE3_12 | (BE3_21)  Frequency of moderate physical activity in past week  1.never 2.1day 3.2days 4.3days 5.4days 6.5days 7.6days 8.daily 9.Don’t know  (BE3_22)  Duration of moderate physical activity  1.<20minutes  2.20-30minutes  3.30-40 minutes  4. 40-50 minutes  5. 50-60 minutes  6. >60 minutes  8. Non-applicable  9. Don’t know  (BE3_11)  Frequency of vigorous physical activity in past week  1.never 2.1day 3.2days 4.3days 5.4days 6.5days 7.6days 8.daily 9.Don’t know  (BE3_12)  Duration of vigorous physical activity  1.<20minutes  2.20-30minutes  3.30-40 minutes  4. 40-50 minutes  5. 50-60 minutes  6. >60 minutes  8. Non-applicable  9. Don’t know | 1.Yes: Participants who were participated in moderate to vigorous aerobic physical activity three to four sessions(frequency) per week and the activity lasted an average of 40 minutes(duration) per sesseion.  0.No: Remainder. |
| Medication adherence | DI1_2 | Regular antihypertensive medication taken(3^rd^)  1.Always regularly  2.Sometimes or only when needed 3.Not taken 8. Non-applicable 9. Don’t know  antihypertensive medication taken(4^th^,5^th^)  1.Daily 2.>20days per a month 3.>15days per a month 4.<15days per a month 5.Not taken 8. Non-applicable | 1.Good: Always regularly (3) or Daily (4,5)  0.Poor: Remainder. |
| Self-reported diet control | N_DIET  N_diet | Diet control (3^rd^)  1.Yes 2.No  Diet control (4^th^,5^th^)  1.Yes 2.No 3.Don’t know/No response | 1.Yes: A participants answered, “yes”  0.No: A participants answered, “No” |
| Sodium intake | N_NA | Sodium intake per day (mg) | Same |
